# Current concepts and practical applications for recovery, growth, and peak performance following significant athletic injury

**DOI:** 10.3389/fpsyg.2022.929487

**Published:** 2022-08-22

**Authors:** Toby J. Brooks, Tyler C. Bradstreet, Julie A. Partridge

**Affiliations:** ^1^Department of Rehabilitation Sciences, School of Health Professions, Texas Tech University Health Sciences Center, Lubbock, TX, United States; ^2^Department of Intercollegiate Athletics, Texas Tech University, Lubbock, TX, United States; ^3^College of Health and Human Sciences, School of Human Sciences, Southern Illinois University at Carbondale, Carbondale, IL, United States

**Keywords:** rehabilitation, recovery, growth, interprofessional care, behavioral medicine, athlete psychology

## Abstract

For decades, physicians, athletic trainers, and other health care professionals have worked to standardize the recovery process following injury to enhance patient outcomes and to help set appropriate goals for return to competition. Traditionally, these efforts have focused primarily on physical and/or physiological aspects of healing with little consideration for psychological aspects. Concurrently, mental health professionals who work with athletes have developed strategies to enhance performance and minimize negative influences of mental aspects of recovery while promoting approaches that include mental as well as physical recovery. Several strategies have emerged that further encourage a multi-faceted and interdisciplinary approach when helping injured patients return to participation. While important in a healthy population, the practical applications of these strategies are likely more critical for an athlete working through the recovery process with an ultimate goal of returning to competition. Despite these realities, both practical experience and a dearth of literature point to the traditional athletic healthcare providers’ common focus on physical aspects of recovery and psychological professionals’ focus primarily on mental aspects has resulted in sub-optimal outcomes compared to the likely benefits of an integrated approach. This article is intended to characterize current concepts in the fields of sport psychology and mental health concerning the importance of mental aspects of recovery in returning to play. Next, the authors will examine how modern theories can influence practice and discuss how these strategies can be effectively integrated and leveraged to enhance recovery and the athlete’s enjoyment of the rehabilitation and ultimately restoration process.

## Introduction

Competitive athletics—particularly at the elite/international level—are frequently viewed by the masses as the very zenith of the human experience. After all, in order for an athlete to achieve or even approach unprecedented levels of human performance, it is likely that all aspects of their preparation have been analyzed, optimized, and analyzed again. Frequently, this involves training and recovery, nutrition, coaching, and tactical aspects of performance. Sport coaches and strength & conditioning specialists develop highly specific plans with the singular goal of maximizing athletic performance. If any aspect of function could be improved, it is likely that the athlete and/or their coaching staff would attempt to leverage that potential to maximum effect.

However, such an approach has traditionally demonstrated a glaring blind spot. Specifically, issues concerning mental health and psychological strategies to enhance performance have historically been lacking relative to their physical and physiological counterparts. The underpinnings of this difference are multivariate and complex, however, most would suggest that (at least until recently) the “warrior mentality” so prevalent in competitive sport has served to stigmatize seeking help for mental health and psychological concerns by the athlete (Glick et al., 2009; Rice et al., 2016). As Olympic medalist Sasha Cohen has adeptly summarized, in the highly competitive realm of elite athletic competition, “You need to show the world that you are strong, so if you were to say, ‘oh, I have mental issues,’ that just cracks the facade of trying to show the world that you’re impervious,” (Rapkin, 2020).

Against this backdrop of historic and systemic resistance to mental and behavioral health care in sport, the medical needs of competitive athletes have often been addressed primarily from a physical rather than more global approach (including mental health care) for decades. At the same time, the pursuit of maximal performance also comes at the price of increased risk of injury. Whether suffered during training or competition, acute or chronic in nature, and managed through more conservative or surgical means, physical injury from sport holds the potential to be a major negative life event. In addition to pain and compromised/lost performance, injuries can also contribute to poor mental health (Gouttebarge et al., 2019; Chang et al., 2020). Unfortunately, given the typical resistance to mental health care even in the healthy state, many athletes may avoid seeking mental health care as a part of the recovery process for physical injury.

To further confound matters, sports medicine professionals (including physicians, athletic trainers and physical therapists) who are commonly present throughout the injury evaluation, care, and rehabilitation process have traditionally lacked the formal academic or clinical preparation in order to appropriately manage mental health concerns in their patients. Such issues are usually considered outside the scope of practice for their respective professions, making referral the only possible intervention for providers of affected patients. However, with the overwhelming majority of sport governing bodies, professional teams, and athletics programs only recently beginning to provide the necessary funding to provide ready access to trained mental health professionals, the typical sports medicine provider is ill prepared and lacking the outside resources to provide or coordinate care to help the injured athlete through this critical aspect of recovery.

Thankfully, over the course of the past two decades, this reality has slowly started to change for the better. Sport organizations and institutions have begun to recognize the critically important nature of a more holistic approach to care, particularly with high profile athletes such as Simone Biles and Naomi Osaka speaking candidly about their experiences with mental health professionals in the recent past. In order to positively impact overall health of athletes through the purposeful inclusion of mental health professionals, administrators and decision makers have allocated the necessary resources to support these efforts. This article has been written to inform the reader regarding the history, current state, and the future of this contemporary interprofessional and more holistic approach to athletic health care. It is hoped that over time, the pioneering programs of the past decade in particular will become the norm rather than the exception.

## Historical perspectives

### The traditional model of athletic health care

While competitive athletics have existed globally since the times of the ancient Greeks and Romans, health care for athletes has varied widely over the centuries. Historical accounts of well-known medical pioneers such as Hippocrates and Galen date some of the earliest known care rendered specifically to athletes as early as 400 BC (Prentice, 2021). However, with the fall of the Roman Empire just prior to 400 AD, interest in sport—and concurrently, the medical needs of athletes—fell dramatically. Not until the Renaissance beginning in the 14th century did interest in sport begin to return.

With the resurgence of sport came a renewed need to provide medical care for athletes. The profession of athletic training, an allied health profession composed of credentialled professionals who function primarily to prevent, recognize, and manage injuries to athletes and the physically active, was first organized to provide for the emerging medical needs of collegiate athletes in the United States in the early 1900s (Ebel, 1999). At the same time, other health care professions have also grown, with many further developing subspecialities specific to rendering care specifically to athletes and the physically active. “Sports medicine” has historically been used as an umbrella term that includes any medical professional who works specifically with athletes, including physicians, physician assistants, nurses and nurse practitioners, athletic trainers, physical therapists, occupational therapists, chiropractors, and other related care givers.

Despite significant growth in the field and the dramatic increase in medical services and coverage for athletes—particularly at the collegiate, professional, and secondary school levels—most efforts since the earliest development of sports medicine have been focused on addressing physical aspects of injury with little to no attention to mental and behavioral aspects of care. While true in all aspects of care, the purpose of this article is to specifically consider the rehabilitation process following significant injury. For many athletes, weeks or even months are spent on physical restoration. However, what is commonly lacking is a more holistic approach to healing that includes mental health care.

As sports medicine has grown over the decades, other professions have emerged as critical components of a seemingly ever-growing team. Strength and conditioning specialists, data analysts, sports nutritionists, and sport psychologists are frequently employed by many professional and upper-level collegiate programs. This more specialized interprofessional model not only ensures each member functions legally within the respective profession’s scope of practice, it also provides for a better level of care than would be possible under more antiquated models of delivery.

### Current and emerging models of integrated athletic health care

The following depicts three of the most common approaches to providing mental health and sport psychology services to competitive athletes following significant injury. It is important to note that all models are not exclusively employed following injury and can be highly valuable in assisting with a variety of peak performance concerns. While some programs continue to offer little to no care (typically due to budget concerns), thankfully, the prevalence of such arrangements is on the decline.

#### In-house programs

Although the costliest alternative, many sport organizations or departments have dedicated full-time positions to one or more sport psychologists to render care to athletes. The potential upside to such programming is tremendous. Athletes can be provided with greater depth and breadth of care and coordination between other members of the sports medicine team is greatly facilitated. Some have noted that the in-house model is important simply because it signals a culture that embraces the importance of mental health, and at the same time serves to de-stigmatize commonly cited resistance to seeking care (Chew and Thompson, 2014). Particularly with respect to the rehabilitating athlete, this model promises to best facilitate communication and interprofessional teamwork among all members of the sports medicine team.

On the other hand, this model does present a few drawbacks. Cost is certainly a concern, however, others have cited limited professional support, high demands on a single clinician, limited resources beyond salary, and potential pressures from those in the notoriously high-speed, high-demand world of athletics being chief among them (Chow et al., 2020). That said, many programs have recognized the critical importance of mental health care as well as the need to “keep up” with care that is being provided in other sport organizations and have either already moved or are making plans to move to this model soon.

#### Counseling centers

Perhaps one of the most convenient means of providing similar services—at least in the collegiate setting—is through the use of the on-campus counseling center. Leveraging the staff and resources of an established facility intended for the general student population can be efficient and cost-effective while at the same time operating free from any potential pressures from athletics department personnel. Many centers include multiple staff members, further facilitating referral or peer consultation for difficult consultation in a way not possible for the common single provider working in athletics (Chew and Thompson, 2014).

Conversely, perhaps the biggest drawback to this model is the lack of consistent preparation among clinicians to work specifically with the athletic population. This can make coordination with other members of the sports medicine team difficult and at times inefficient. Additionally, timeliness of care may not be in line with the typical expectations in the high-pressure athletics environment. Lastly, while this may be a viable option on a large college campus, professional sports teams, small colleges, secondary schools, and other private sport organizations are unlikely to have access to such arrangements.

#### Outside consultants

Lastly, the final option (other than no provision at all) for providing mental health care services to athletes is through the use of one or more part-time consultants. Like the previous two models, this arrangement has its own inherent strengths and weaknesses. This approach could be as involved as a pre-negotiated baseline of hours provided to the sports medicine team or as informal as an identified professional in the community who will serve as a referral resource on an as-needed basis.

While certainly better than no care at all, this approach can make consistency of care a challenge. For example, some institutions may have arrangements with one sport psychologist for one athletic team and another for others. Services may be limited based on the expertise of the provider and barriers such as transportation to and from the clinic may complicate coordination of care. This model may also seem to perpetuate the notion that mental health concerns are uncommon and not dealt with frequently enough to warrant a more formal arrangement, further perpetuating the stigma associated with seeking care. Particularly for the athlete recovering from significant injury, this model is characteristically inefficient and cumbersome relative to other approaches.

## Current theoretical concepts

In order to understand the impact of injury on athletes’ psychological well-being, several different theories have been developed, two of which are discussed below.

### Integrated sport injury model

The injury process is dynamic, variable, and athlete-specific. Understanding the psychological impact of an injury—how an athlete responds to injury and rehabilitation—is pivotal to a holistic recovery process. Wiese-Bjornstal and colleagues (Wiese-Bjornstal et al., 1998) proposed the Integrated Sports Injury Model (ISI), which is the most accepted and well-developed model to date (Brewer, 2001). It is beyond the scope of this paper to describe the model in full; however, in brief, the ISI encompasses (a) the impact of biopsychosocial variables on the stress response and the likelihood of injury onset, (b) the personal and situational influencing factors, and (c) the cognitive, emotional, and behavioral responses to the injury during the rehabilitation process (Table 1).

**TABLE 1:** Integrated sport injury model.

Aspect of response	Examples
Pre-injury factors	locus of control, goal orientation, motivation, trait anxiety, history of stressors, coping skills
Personal and situational factors	pain tolerance, athletic identity, injury severity, competition level, social support, mental status
Cognitive, emotional, and behavioral response	self-esteem, rate of perceived recovery, grief, fear of the unknown, rehab adherence, effort

The foundational component of the ISI is the resulting cognitive appraisals due to the interaction between the precipitating stress response that led to the injury, situation-specific factors, and person-specific factors. Consistent with Beck’s cognitive model (Beck and Haigh, 2014), these cognitive appraisals drive the athlete’s subsequent emotional and behavioral responses. If the cognitive appraisals are adaptive in nature, the athlete is more likely to head toward full recovery, but if they are maladaptive, then a downward spiral away from full recovery could occur. Ultimately, the ISI demonstrates that the cognitive appraisal process is critical to ensuring successful psychosocial and physical recovery outcomes post-injury.

### Sport injury related growth

While a successful injury rehabilitation process is crucial, it is important to consider how we can harness that time to not only help the athlete return to their pre-injury baseline ability but also leverage it as an experience of personal growth to aid in even further wellness and development. In fact, researchers have suggested that after enduring the challenge of a long rehabilitation period, many athletes report being more dedicated, focused, and mentally and physically stronger than they were pre-injury (Wiese-Bjornstal et al., 1998). This is demonstrated in the work by Walker and colleagues (Walker et al., 2007), who expanded on the ISI by increasing insight on the importance of healthy individualized meaning-making throughout the injury experience.

Roy-Davis and colleagues coined the term Sport Injury Related Growth (SIRG) to reflect the multi-faceted development that can be cultivated during recovery form sport injury (Roy-Davis et al., 2017). In brief, SIRG suggests that injured athletes who have certain characteristics (e.g., optimistic explanatory style, access to appropriate resources such as a rehabilitation specialist, previous experiences of adversity to draw upon, emotion- and problem-focused coping styles, and a strong social support system) are more likely to experience positive adaptations and growth post-injury. Specifically, harnessing these characteristics, skills, and resources leads athletes to (a) be more aware of and have control over their thoughts, (b) have greater cognitive reappraisal abilities to view experience as a developmental opportunity, (c) experience positive emotions, and (d) engage in facilitative actions, which in turn lead to positive adaptation and growth in social, mental, physical, and sport-specific domains (Wadey et al., 2020). Recent research has proposed a five-dimension model to describe SIRG in athletes (Table 2; Rubio et al., 2020).

**TABLE 2:** Dimensions of sports injury related growth (SIRG).

Dimension	Examples
Personal strength	increased empathy, mental toughness, self-reflection, hardiness, optimism, and resilience
Improved social life	improved appreciation for and fostering of relationships and being a member of a team
Health-related benefits	enhanced pain management, awareness of injury prevention, commitment to maintaining health
Sport-related benefits	found a more valuable role on team, worked on technical skills, developed greater mental skills
Social support and recognition	was valued by others beyond the athlete role, received support for needs and responsibilities

## Practical applications

### Integrated health care

Over the last two decades, Integrated Health Care (IHC) has become a commonly accepted practice to help health care become less fragmented and to shift the focus to treating the whole person from a biopsychosocial standpoint and produce greater patient outcomes in a value-based manner (McDaniel and deGruy, 2014; World Health Organization, 2016). In essence, IHC serves as an overarching term for a dynamic set of principles that seek to provide greater healthcare around the totality of a patient’s needs. Within sports medicine, IHC can be seen in the ever-increasing athlete-centered medical homes and the commitment to multidisciplinary physical and mental health (Courson et al., 2014).

This commitment to IHC can be seen in sports injury rehabilitation as well. In addition to the ISI model previously discussed, there have been several other theoretical lines of research that demonstrate the importance of the psychology of injury on a successful rehabilitation process. Personal Investment Theory (Duda et al., 1989) posits that motivation during rehabilitation is determined by personal incentives, sense of self, and perceived options to accomplish recovery and growth. For example, if an athlete sees rehabilitation as a way to get back with the team and satisfy their social needs, believes they are competent and skilled in their sport, and has trust in their rehabilitation specialist(s), they are more likely to be an active participant in their recovery.

Protection Motivation Theory (Floyd et al., 2000) suggests that injury rehabilitation adherence is influenced by the severity and susceptibility of the injury and how effective the patient perceives the intervention(s) to be along with their ability to reliably implement it. In other words, a patient’s adherence depends upon factors including self-efficacy, available coping skills, and management of any threat appraisals that arise during the process. Additionally, the Sport Injury Rehabilitation Adherence Model (Brewer, 1998) offers key factors that have the most impact on an athlete’s adherence level. For example, (a) behaviors such as compliance and enthusiastic engagement with the rehabilitation plan, (b) personal factors such as intrinsic motivation, cognitive flexibility, and task and mastery goal orientation, and (c) positive beliefs surrounding the rehabilitation process stemming from social support, comfort and convenience of the rehabilitation program and setting, and trust in the rehabilitation specialist(s) all lead to greater adherence throughout the process. Overall, these models continue to demonstrate the importance of the consideration and implementation of psychological skills and interventions within the athletic injury rehabilitation process.

### Psychological interventions

While there are an array of effective psychological skills that have been found to aid and facilitate recovery and growth during the injury rehabilitation process, robust evidence is lacking due to inter-athlete variability. However, a limited number of meta-analyses have been published to date regarding general psychological interventions with injured athletes (Ivarsson et al., 2017; Zakrajsek and Blanton, 2017; Gledhill et al., 2018; Gennarelli et al., 2020; Li et al., 2020). In general, these works identified the following interventions to be effective for promoting recovery after a sports injury: imagery, relaxation, goal setting, positive self-talk, coping skills, modeling, psychoeducation, biofeedback, and social support. Likewise, the implementation of these interventions produced positive mood changes, improved self-efficacy, reduced stress and anxiety, improved motivation and satisfaction, healthier cognitive appraisals, more effective pain management, enhanced exercise compliance, and overall improved rehabilitation adherence, suggesting that they are effective and beneficial for injured athletes.

Beyond these individual psychological skills, there are three relatively commonly employed and evidence-based psychological interventions that have not only demonstrated effectiveness in positively impacting the injury rehabilitation process but are used across a wide-array of mental and behavioral health concerns. These include Mindfulness-Based Stress Reduction Training (MBSR), Acceptance and Commitment Training (ACT), and Motivational Enhancement Training (MET). Each is a manualized treatment that can also be adapted in various ways as applied interventions.

#### Mindfulness-based stress reduction training

First, MBSR focuses on strategies grounded in mindfulness meditation as a self-regulatory approach to stress reduction and cognitive and emotional management (Kabat-Zinn, 2003). MBSR’s primary components include education on mindfulness and meditation practices that incorporate various breathing and cognitive techniques. As a standardized protocol, MBSR is an eight-week stress reduction program that starts with a group retreat as an introduction to mindfulness meditation, weekly group sessions focused on meditation practice, group discussion, mindfulness skill-building activities, and daily individual meditation practice. During the injury rehabilitation process, athletes who complete MBSR have demonstrated increased pain tolerance, increased frequency of daily mindful states, and reductions in psychological distress (Mohammed et al., 2018). Beyond MBSR, other mindfulness-based interventions have also been utilized with athletes, such as Mindfulness Sport Performance Enhancement (MSPE) (Kaufman et al., 2018). In general, mindfulness as a foundational component can also be pulled out as an intervention, as mindfulness has been associated with greater self-regulation *via* metacognitive awareness, decreased cognitive rumination and emotional reactivity, increased cognitive flexibility, and greater attentional capacity (Davis and Hayes, 2011; Tang et al., 2015; Guendelman et al., 2017).

#### Acceptance and commitment training

Acceptance and commitment training (ACT) focuses on strategies aimed at increasing psychological flexibility (Hayes et al., 2012). Much like MBSR, the foundational component of ACT is mindfulness along with the added component of committed action that is grounded in core values rather than goals. More specifically, ACT has six core therapeutic processes: (1) being psychologically present, (2) noticing but not getting caught up in our thoughts, (3) opening up and making room for our feelings, (4) understanding that we are not our actions, feelings, or thoughts, (5) knowing our values, and (6) engaging in intentional behavior that is guided by our actions. While there have been few treatment studies on ACT during sport injury rehabilitation, insights that can be gleaned from the literature, in general, demonstrate the likelihood of effectiveness and its relationship with the central tenants of ISI and SIRG.

Acceptance and commitment training has been considered a unified model of behavior change given that it is transdiagnostic, process-focused, and flexible with broad applicability (Dindo et al., 2017). ACT has been found to lead to improved mood states, decreased anxiety, greater quality of life, and more effective management of fatigue and chronic pain, among others (Hayes et al., 2006). The primary mechanisms within ACT that lead to change and improved outcomes across an array of clinical presentations are increasing psychological flexibility and decreasing experiential avoidance (Stockton et al., 2019).

#### Motivational enhancement training

Motivational Enhancement Training (MET) focuses on strategies to strengthen personal motivation for and commitment to a specific goal by exploring the person’s own reasons for change within an atmosphere of acceptance and compassion (Miller and Rollnick, 2013). The foundational component of MET is motivational interviewing, which is a collaborative, goal-oriented style of communication that pays particular attention to the language of change. It is designed to strengthen personal motivation for and commitment to a specific goal by eliciting and exploring the person’s own reasons for change within an atmosphere of acceptance and compassion (Rollnick et al., 2010). The five principles of MET include expressing empathy *via* reflective listening, developing the discrepancy between current and future self, rolling with the resistance and diverting or directing toward positive change, avoiding argumentation, and supporting self-efficacy. The four processes of MET include engaging and building a foundational relationship, defining and focusing the discussion on the target of change, evoking reasons and abilities for change, and building commitment to change and planned action.

Much like ACT, while there have been relatively few treatment studies on MET focused on sport-related settings, early findings suggest that the technique is effective and can help keep the patient sufficiently motivated and engaged in the rehabilitation plan. Generally speaking, MET has been found to lead to improved patient outcomes across a broad range of physical and mental health issues, particularly in part due to stopping or preventing health-interfering behaviors and engaging in health-promoting behaviors (Rollnick et al., 2008). The primary mechanism within MET that leads to more productive behaviors is establishing intrinsic motivation for change to overcome ambivalence (Csillik, 2015). Practically, motivational interviewing is often used as a technique in and of itself that gets combined with other cognitive and behavior therapies, such as ACT.

### Creating an integrated health care rehabilitation environment

A commitment to IHC and the biopsychosocial mechanisms that impact the injury rehabilitation process is necessary for SIRG. Healthcare professionals working with athletic populations—particularly with a focus on injury rehabilitation—must find ways establish an integrated rehabilitation environment grounded in the ISI. There are two primary mechanisms to accomplish this task. First, we must expand our conception of sports medicine and ensure the presence of a mental health professional on the multidisciplinary treatment team. Doing so allows for more effective and immediate psychological interventions necessary for recovery and post-injury growth. Second, we must ensure that medical health professionals are adequately trained in relevant psychology of injury theories and related psychological interventions (as appropriate). This can serve as a prevention method to mitigate potential adherence issues, can augment and reaffirm the work of the mental health professional with the athlete, or simply help the medical health professional provide more appropriate care if there is not an ability to have a mental health professional on the treatment team. Additionally, given the role of the environment on the injury rehabilitation process, we must provide education to the entire athletic system and social support network around the injured athlete. Such inclusive education can most assuredly help foster a greater injury rehabilitation process, spur on post-injury growth, and increase the likelihood of peak performance upon return to play.

## Future directions

Injury rehabilitation is a crucial component of the sport realm, and advancements in our understanding of not only the physical, but also psychological/mental/emotional aspects of the recovery process continue to evolve. Integration of a comprehensive and qualified sports medicine team can and should be considered as a standard of care to which all athletes are entitled. Furthermore, systematic and rigorous research should be utilized to increase our understanding of the psychological rehabilitation process to better inform how practitioners can provide the most effective and holistic response to an injury event.

## Author contributions

TJB served as first author of the work and contributed to the conceptualization, initial drafting, revision, and final formatting of the manuscript. TCB served as second author of the work and contributed to the conceptualization, initial drafting, and revision of the manuscript. JAP served as third author of the work and contributed to the conceptualization, initial drafting, and revision of the manuscript. All authors contributed to the article and approved the submitted version.
